# TGF-β2 enhances expression of equine bone marrow-derived mesenchymal stem cell paracrine factors with known associations to tendon healing

**DOI:** 10.1186/s13287-022-03172-9

**Published:** 2022-09-16

**Authors:** Drew W. Koch, Lauren V. Schnabel, Ilene M. Ellis, Rowan E. Bates, Alix K. Berglund

**Affiliations:** 1 Department of Clinical Sciences, College of Veterinary Medicine, North Carolina State University, Raleigh, NC USA; 2 Comparative Medicine Institute, North Carolina State University, Raleigh, NC USA

**Keywords:** Mesenchymal stem cell, Tenocyte, TGF-β, Equine, Tendon, RNA-sequencing, Paracrine factors, Extracellular matrix

## Abstract

**Background:**

Mesenchymal stem cells (MSCs) secrete paracrine factors and extracellular matrix proteins that contribute to their ability to support tissue healing and regeneration. Both the transcriptome and the secretome of MSCs can be altered by treating the cells with cytokines, but neither have been thoroughly investigated following treatment with the specific cytokine transforming growth factor (TGF)-β2.

**Methods:**

RNA-sequencing and western blotting were used to compare gene and protein expression between untreated and TGF-β2-treated equine bone marrow-derived MSCs (BM-MSCs). A co-culture system was utilized to compare equine tenocyte migration during co-culture with untreated and TGF-β2-treated BM-MSCs.

**Results:**

TGF-β2 treatment significantly upregulated gene expression of collagens, extracellular matrix molecules, and growth factors. Protein expression of collagen type I and tenascin-C was also confirmed to be upregulated in TGF-β2-treated BM-MSCs compared to untreated BM-MSCs. Both untreated and TGF-β2-treated BM-MSCs increased tenocyte migration in vitro.

**Conclusions:**

Treating equine BM-MSCs with TGF-β2 significantly increases production of paracrine factors and extracellular matrix molecules important for tendon healing and promotes the migration of tenocytes in vitro.

**Supplementary Information:**

The online version contains supplementary material available at 10.1186/s13287-022-03172-9.

## Background

Mesenchymal stem cells (MSCs) are fibroblast-like cells that can be isolated from various tissues including bone marrow, adipose, peripheral blood, and cord blood. Although MSCs can be directed to differentiate into cartilage, fat, and bone in vitro, there is little evidence to support that MSCs engraft and differentiate into new tissue once injected in vivo [1]. Instead, MSCs secrete numerous paracrine factors that recruit and support the differentiation of endogenous progenitor cells, promote angiogenesis, inhibit apoptosis, and modulate local immune responses [2]. Extracellular matrix (ECM) proteins secreted by MSCs may also directly contribute to tissue healing or be used to generate acellular scaffolds for tissue engineering [3–5]. MSCs are therefore of interest as a regenerative therapy to enhance the endogenous healing of injured tissues.

Due to the lack of inherent healing capacity within the tendon, improved therapies for tendon regeneration are needed to promote return to functionality after injury. Bone marrow-derived MSCs are currently used to treat tendon injuries in horses and are actively being investigated in preclinical research and clinical trials for treating human tendon injuries as well. Horses with superficial digital flexor tendon (SDFT) injuries treated with autologous MSCs had reduced reinjury rates, improved fiber alignment, and less inflammatory cell infiltrate compared to horses that did not receive MSCs [6–8]. In a rat Achilles tendon repair model, tendons treated with BM-MSCs had higher maximum load to failure and stiffness compared to control tendons as well as increased expression of pro-tendon healing factors, scleraxis and tenomodulin [9]. However, results from human clinical trials using MSCs to treat musculoskeletal injuries have failed to meet the expectations set by preclinical animal models. A number of factors have likely contributed to this including immunocompatibility, cell quality and dose, and inconsistent culture protocols [10–12]. Current trends in MSC research are therefore focused on improving the consistency and quality of MSCs to increase their therapeutic efficacy.

There is considerable interest in in vitro priming or “licensing” of MSCs to enhance their secretome and increase the expression of paracrine factors specific for the type of injury or disease being treated. Previous research has focused primarily on priming MSCs with inflammatory cytokines like IFN-γ, TNF-α, or IL-1β to increase expression of growth factors and anti-inflammatory cytokines from MSCs [13]. We previously published that allogeneic equine BM-MSCs cultured with TGF-β2 have reduced MHC I surface expression and cell-mediated cytotoxicity in vitro while retaining their abilities to suppress T cell proliferation and secrete PGE2 and TGF-β1 [14]. However, TGF-β is known to affect the expression of other cytokines, growth factors, and extracellular matrix molecules that may affect the MSC functions and the efficacy of MSC therapy [15, 16].

The purpose of this study was to determine how TGF-β2 changes the transcriptome of MSCs with an emphasis on secreted factors relevant to tendon healing in equine BM-MSCs. Through use of RNA-sequencing, western blotting, and a tenocyte migration assay, we discovered that TGF-β2-treated BM-MSCs have enhanced expression of extracellular matrix molecules including collagen and tenascin-C and, similar to untreated MSCs, increase tenocyte migration, all of which may improve their therapeutic efficacy for in vivo tendon healing.

## Methods

### Animal use and welfare

A total of 13 systemically healthy horses were utilized in this study for sternal bone marrow harvest as approved under protocol #19–628 for sedated bone marrow collection or #20–454 for postmortem collection by the Institutional Animal Care and Use Committee of North Carolina State University. All horses were between the ages of 6–16 years of age, were Thoroughbred or Thoroughbred crosses, and an equal number of mares and geldings were used in each experiment. The SDFT obtained for tenocyte isolation was collected from a 16 year old Thoroughbred horse euthanized for reasons unrelated to this study as approved under protocol #20–454.

### MSC isolation and culture

Bone marrow aspirates were collected aseptically from the sternum of horses using 11-gauge Jamshidi bone marrow aspirate biopsy needles under standing sedation with local lidocaine anesthesia or immediately post-euthanasia. Bone marrow aspirates were purified via Ficoll-Paque Plus (GE Healthcare) gradient centrifugation. Isolated cells were cultured in standard media containing 1 g/dl glucose DMEM (Corning), 10% fetal bovine serum (Atlanta Biologicals), 2 mM L-glutamine, 100 U/ml penicillin and streptomycin, and 1 ng/ml recombinant human basic fibroblast growth factor (bFGF)(Corning). When cultures reached 80% confluency, MSCs were passaged with Accutase cell-dissociation solution (Innovative Cell Technologies) and plated at a density of 6,250 cells per cm^2^ on 100 mm tissue culture plates. BM-MSCs were passaged for 2 to 4 passages prior to use. Cell count and viability at each passage were determined using a Cellometer® Auto 2000 and ViaStain™ AOPI Staining Solution (Nexcelom Bioscience LLC). BM-MSCs were treated by adding 1 ng/ml recombinant human TGF-β2 (BioLegend) to the media as previously described for 3 passages prior to RNA-sequencing or for 72 h for all other analyses [17]. We have previously published that MSCs isolated and cultured using this protocol are positive for CD29, CD44, and CD90 and negative for LFA-1 and CD45 [17].

### RNA-sequencing

Total RNA was extracted from paired P3 or P4 untreated and TGF-β2-treated BM-MSCs from four horses using the RNeasy Mini kit (Qiagen). Libraries were generated and poly(A) enriched using 1 ug of RNA as input. Indexed samples were sequenced using a 150 base pair paired-end protocol on a HiSeq 2500 (Illumina) according to the manufacturer’s protocol. Sequence reads were trimmed to remove possible adaptor sequences and nucleotides with poor quality using Trimmomatic v.0.36. The trimmed reads were mapped to the EquCab 3.0 using the STAR aligner v.2.5.2b. Unique gene hit counts were calculated using featureCounts from the Subread package v.1.5.2. Using DESeq2, a comparison of gene expression between the untreated and TGF-β2-treated BM-MSCs was performed. The Wald test was used to generate p values and log2 fold changes. Genes with an adjusted p value of < 0.05 and log_2_ fold change > 1 were determined to be differentially expressed for each comparison. The quantification and poly(A) selection of mRNA, library preparation, sequencing, and bioinformatics were outsourced to GENEWIZ, Inc.

### Protein lysate collection and western blotting

BM-MSCs from four additional horses not used for RNA-sequencing were cultured on 100 mm tissue culture plates in control media or media containing 1 ng/ml TGF-β2 for 72 h. The plates were washed with ice cold phosphate buffered saline (PBS) before adding 350 µl of cold RIPA Lysis and Extraction Buffer (Thermo Scientific) with 1X Halt Protease Inhibitor Cocktail (Thermo Scientific). Plates were scraped on ice and the contents transferred to a microcentrifuge tube and incubated on ice for 15 min. Microcentrifuge tubes were then centrifuged at 14,000* g* for 10 min. The protein lysate supernatant was collected and frozen at – 80 °C until used. For western blotting, 20 µg of protein lysate was boiled at 100 °C for 5 min with 1X Laemmli buffer and 5% 2-mercaptoethanol and resolved using an Any kD Criterion TGX gel (Bio-Rad). Protein was transferred to a PVDF membrane using a Trans-Blot Turbo Blotting System (Bio-Rad). The membrane was blocked with 5% non-fat dry milk before blotting the membrane with rabbit anti-human type I collagen antibody (Novus Biologicals, NBP168942) or rabbit anti-human tenascin-C antibody (Millipore Sigma, AB19011) [18] both at 1:500 in tris-buffered saline with 0.1% Tween-20 detergent. A mouse anti-human beta-actin antibody was used as a loading control at 1:10,000 (Invitrogen, clone 15G5A11/E2). The membrane was probed overnight at 4 °C before adding mouse anti-rabbit IgG-HRP secondary antibody (Cell Signaling Technology) at 1:2000 or goat anti-mouse IgG-HRP secondary antibody (Invitrogen) at 1:2000 for 1 h at room temperature. The membrane was incubated with Clarity Western ECL Substrate (Bio-Rad) for 5 min before imaging. Images were captured by a luminescent image analyzer with a CCD camera (Bio-Rad). Quantification of expression levels was calculated using ImageJ software (NIH), and values were expressed in arbitrary units relative to beta-actin (ACTB).

### MSC and tenocyte co-culture

To determine the effects of paracrine factors from TGF-β2-treated BM-MSCs on tendon, tenocytes were isolated from a forelimb equine SDFT similar to previously published methods [19]. The SDFT palmar to the third metacarpal bone was aseptically dissected and the tendon was transported in sterile PBS with 100 U/ml penicillin and streptomycin and transferred to a tissue culture hood. Under aseptic conditions, 5 mm × 5 mm × 5 mm tendon explants were created ensuring the center of the SDFT was isolated and paratenon excluded. Explants were digested for 12–18 h in media containing 1 g/dl glucose DMEM (Corning) supplemented with 10% fetal bovine serum (Atlanta Biologicals), 100 U/ml penicillin and streptomycin, 2 mM L-glutamine, 20 µg/ml α-ketoglutaric acid, and 50 µg/mL ascorbic acid and 0.3% collagenase type I (Gibco). Following digestion, media was filtered through a 100 µm Nylon cell strainer before centrifuging the cell solution at 800 g. Viability and cell count were obtained, and cells were cultured on 100 mm tissue culture plates at 8800 cells per cm^2^ in tenocyte media (as previously mentioned, without collagenase). Tenocytes were passaged with trypsin–EDTA (0.25%) when plates became confluent and subcultured to P2 prior to co-culture.

Untreated or TGF-β2-treated BM-MSCs from five additional horses and on horse also used for western blotting were seeded onto Transwell® polycarbonate membrane inserts (MilliporeSigma) in tenocyte media at a density of 20,000 cells per cm^2^. Tenocytes were similarly plated in Transwell inserts at 20,000 cells per cm^2^ as a control and to standardize nutrient deprivation between treatment groups. Cells were allowed 24 h to attach on inserts before adding to scratch wound wells for co-culture. Tenocytes were plated at a density of 6,250 cells per cm^2^ in the wells of a 12-well tissue culture plate and allowed to grow to confluency. Twenty-four hours prior to tenocyte wounding, tenocytes were transitioned to serum-free tenocyte media. A single vertical scratch wound was created in each well using a 200 µl pipette tip. The media was then aspirated, wells were washed twice with PBS, and serum-free tenocyte media was replaced. Images were taken at baseline and then every 12 h for 48 h at three separate locations along each scratch wound using an IX83 inverted microscope and cellSens™ software (Olympus). All images were obtained with the Transwell inserts removed, and to ensure the same location was imaged at each time point, a plate map was created and saved within cellSens™ software. Using Adobe Photoshop, the pixel area of the scratch wound at each location for all treatment groups was measured at baseline (time 0) and then each subsequent time point. The percent closure was calculated by averaging the three locations for each treatment group at each time point using the equation ((Average Area Time 0 − Average Area Time x)/Average Area Time 0)) × 100 where “x” represents time.

### Statistical analysis

The functional enrichment analysis of RNA-sequencing data was performed using g:Profiler (version e104_eg51_p15_3922dba) with g:SCS multiple testing correction method applying significance threshold of 0.05 [20]. Western blot data were normalized by log transformation and analyzed using paired one-tailed t tests with a null hypothesis of no difference using JMP Pro 15 (SAS Institute Inc.). The percent wound healing data for the tenocyte scratch assays were analyzed using a one-way repeated measures ANOVA followed by a Tukey’s test for multiple comparisons using Prism 9.0 (GraphPad).

## Results

### Differentially expressed genes in TGF-β2-treated BM-MSCs

**Fig. 1:**
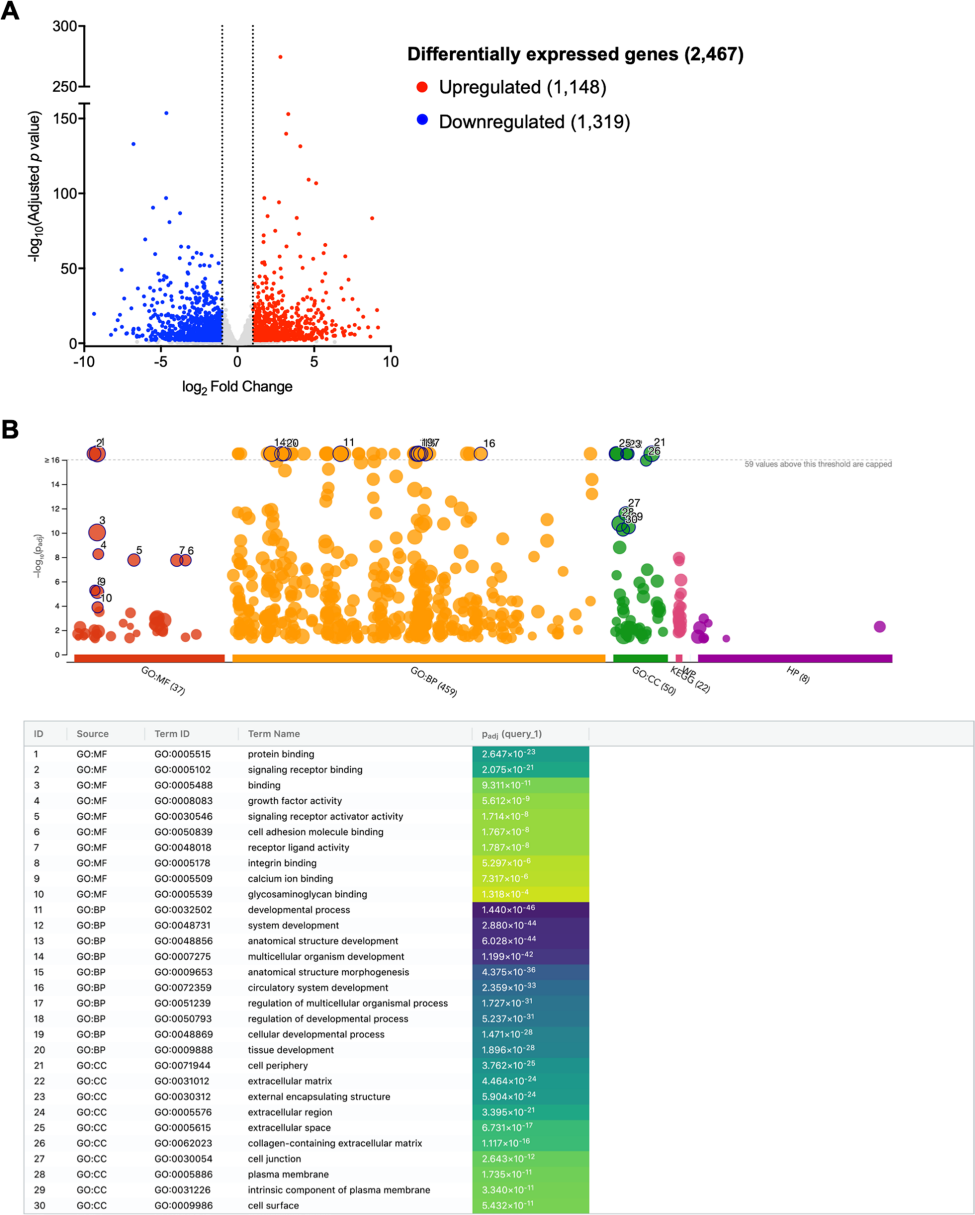
RNA-sequencing and functional enrichment analysis of differentially expressed genes (DEGs). **a** Volcano plot depicting the log_2_ fold change and -log_10_(adjusted *p* value) for gene transcripts in TGF-β2-treated equine bone marrow-derived MSCs detected by RNA-sequencing. Significantly upregulated DEGs are shown in red and significantly downregulated DEGs in blue. **b** Manhattan plots from g:Profiler illustrating GO term enrichment analysis of DEGs in TGF-β2-treated equine MSCs. The top 10 enriched pathways for each GO term (MF: molecular function; BP: biological process; CC: cellular component) are listed below

To identify global changes in gene expression after TGF-β2 treatment, RNA-sequencing was performed on matched untreated and TGF-β2-treated BM-MSCs from four donor horses. We observed 2467 significant differentially expressed genes (DEGs) in total with 1148 genes upregulated and 1319 genes downregulated (Fig. 1a). The genes with the largest increase in expression were INHBE, a member of the TGF-β superfamily, IFGBP5, an insulin-like growth factor binding protein, and RELN, a glycoprotein involved in neuronal migration. The genes with the largest decrease in expression were SCARA5, a ferritin receptor, PDE1A, a phosphodiesterase involved in cAMP signaling, and C30H1orf115, an uncharacterized homolog of the human gene C1orf115. Gene ontology (GO) enrichment analysis was performed to identify the top overrepresented terms in the entire DEG set. The top ten molecular functions (MF), biological processes (BP), and cellular components (CC) for this data are listed in Fig. 1b. Among the overrepresented GO terms relevant to tendon healing were extracellular matrix, collagen-containing extracellular matrix, tissue development, cell adhesion molecule binding, and growth factor activity. For the top 200 upregulated genes, all significant GO terms for MF, BP, CC, Kyoto Encyclopedia of Genes and Genomes (KEGG) pathways, and human phenotype ontology (HP) are shown in Additional file 1: Fig. S1.

### Collagen expression is upregulated in TGF-β2-treated BM-MSCs

**Fig. 2:**
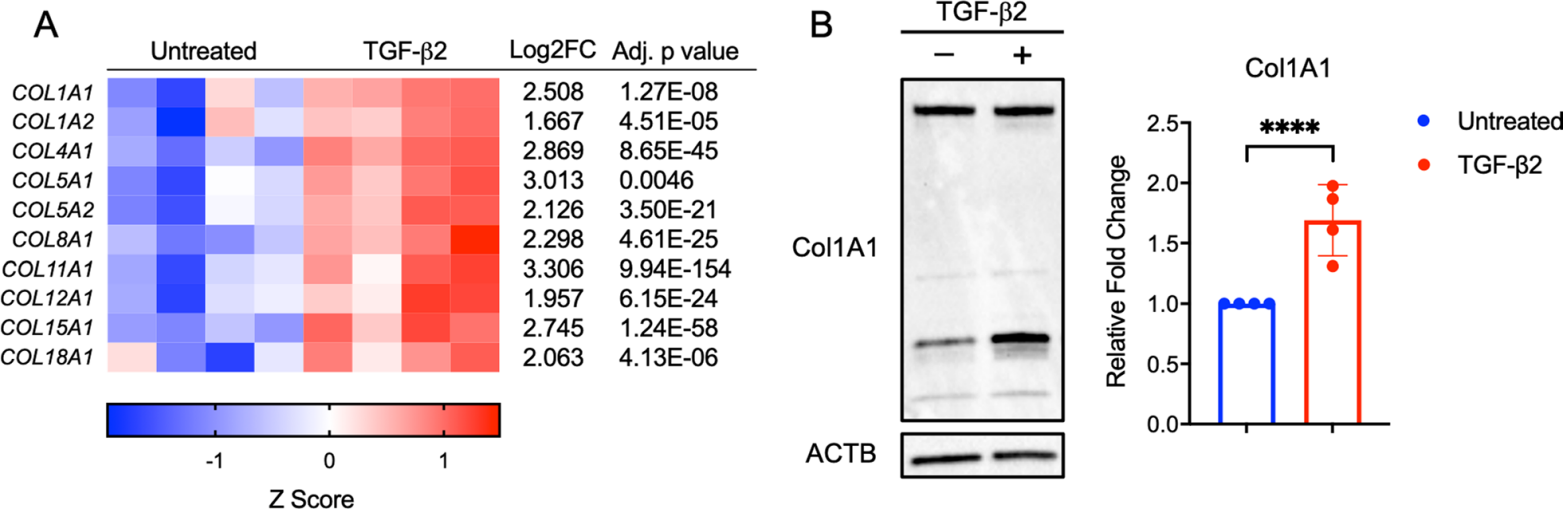
Collagen expression in equine MSCs. **a** Heatmap representation of collagen genes from RNA-sequencing for untreated and TGF-β2-treated equine MSCs. Data shown are Z scores, log_2_ fold change, and the adjusted *p* value for each gene for n = 4. **b** Representative western blot of Col1A1 and ACTB protein expression in untreated and TGF-β2-treated equine MSCs and the densitometric comparison of the relative expression of Col1A1 to ACTB. Data shown are mean ± SD for n = 4, ****p = 0.0001 by a one-tailed t test

Twenty-eight different types of collagens have been described, although types I, II, III, IV, and V are the most common [21, 22]. Normal, healthy tendon is composed primarily of type I collagen. Regenerative therapies for tendons aim to increase type I collagen and limit persistence of type III collagen that is secreted during the proliferative phase of tendon healing, but is structurally weaker. In BM-MSCs treated with TGF-β2, collagen types I, IV, V, VIII, XI, XII, XV, and XVIII were significantly upregulated compared to untreated BM-MSCs (Fig. 2a). COL1A1 and COL1A2 were by far the most abundant collagen transcripts detected in both untreated and TGF-β2-treated equine BM-MSCs followed by COL5A1, COL5A2, and COL12A1. COL11A1, which has been shown to regulate type I collagen fibril assembly [23, 24], had the greatest Log2 fold change in TGF-β2-treated BM-MSCs compared to untreated. Both subunits associated with type V collagen were upregulated, which may support collagen fibrillogenesis [25]. Gene transcripts of type II and type III collagens were not detected by RNA-sequencing. Protein expression of alpha-1 type I collagen from untreated and TGF-β2-treated BM-MSCs from 4 different donor horses was measured by western blot (Fig. 2b). Prominent bands could be detected around both 180 kDa and 35 kDa, which likely correspond to the procollagen alpha-1 chain and procollagen type I carboxy-terminal propeptide, respectively [26, 27]. All horses expressed significantly more total alpha-1 type I collagen from TGF-β2-treated BM-MSCs than untreated (*p* = 0.0001) (Fig. 2b). This significant increase in collagen production by TGF-β2-treated BM-MSCs may be beneficial in improving healing in tissues like tendons where collagen production is critical to the structure, integrity, and function of the tissue.

### Extracellular matrix genes and enzymes are differentially expressed in TGF-β2-treated MSCs

**Fig. 3:**
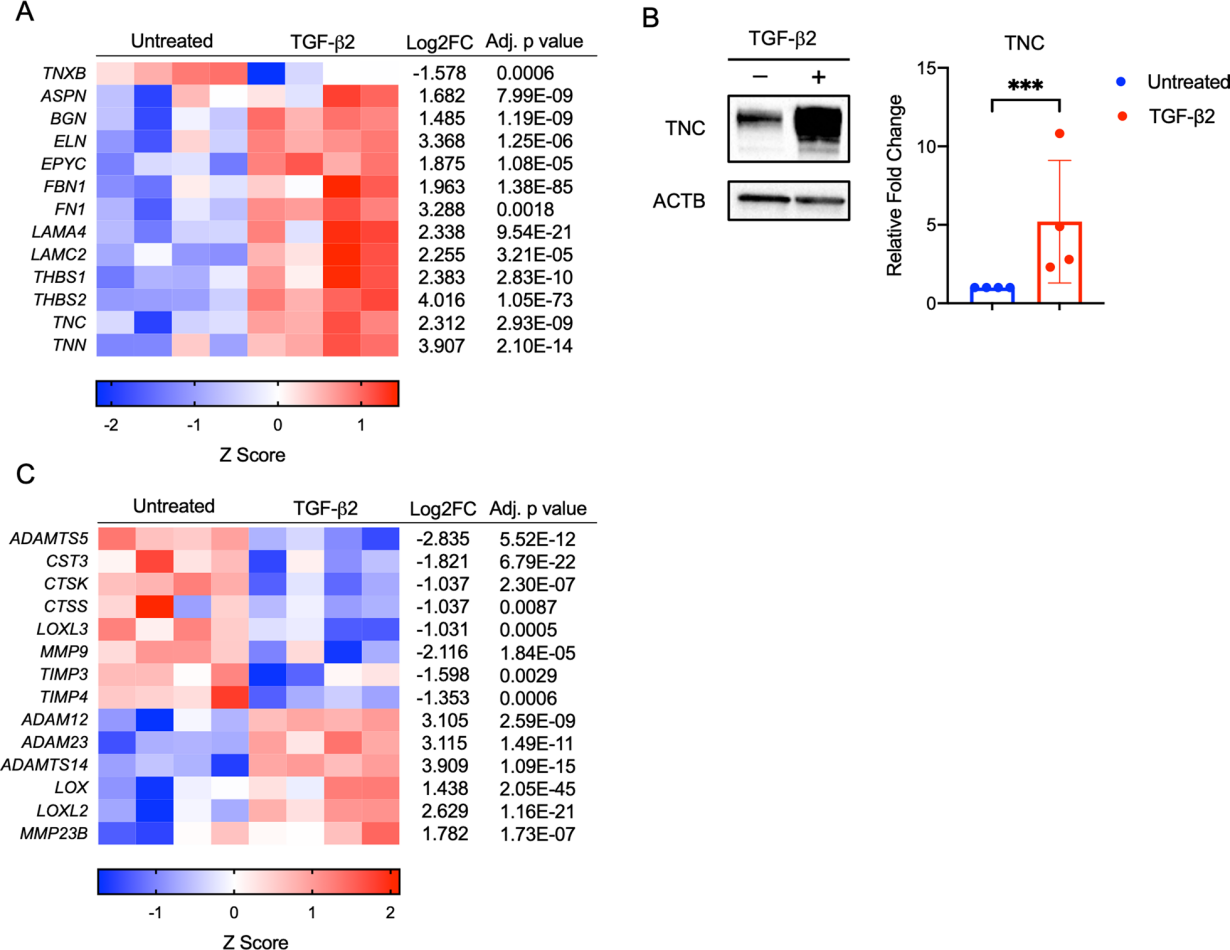
Extracellular matrix protein and enzyme expression in equine MSCs. **a** Heatmap representation of extracellular matrix protein genes from RNA-sequencing for untreated and TGF-β-treated equine MSCs. Data shown are Z scores, log_2_ fold change, and the adjusted *p* value for each gene for n = 4. **b** Representative western blot of TNC and ACTB protein expression in untreated and TGF-β2-treated equine MSCs and the densitometric comparison of the relative expression of TNC to ACTB. Data shown are mean ± SD for n = 4, ***p = 0.0005 by a one-tailed t test. **c** Heatmap representation of extracellular matrix enzyme genes from RNA-sequencing for untreated and TGF-β2-treated equine MSCs. Data shown are Z scores, log_2_ fold change, and the adjusted *p* value for each gene for n = 4

Numerous other non-collagenous ECM genes linked to healthy tendon or tendon healing were upregulated in TGF-β2-treated BM-MSCs including tenascin-C, biglycan, elastin, and fibronectin (Fig. 3a). Tenascin-C is a known marker of tenogenic differentiation and tendon ECM; it plays an important role in cell migration during wound healing, collagen fibril organization, and creates a de-adhesive ECM environment during injury repair [28, 29]. Protein expression of tenascin-C was confirmed to be significantly upregulated in TGF-β2-treated BM-MSCs compared to untreated BM-MSCs (*p* = 0.0005) (Fig. 3b). Of the tendon relevant ECM genes, only tenascin-X (TNXB) was downregulated in TGF-β2-treated BM-MSCs. Along with increases in collagen expression, increases in ECM protein expression from TGF-β2-treated BM-MSCs may assist and enhance tendon repair and healing.

Also important to the ECM in tendon healing are the enzymes that assist with remodeling, collagen cross-linking, and regulation of growth factors. Matrix metalloproteinase-9 (MMP9) is suggested to play a role in early tendon remodeling [30] and was downregulated in TGF-β2-treated BM-MSCs (Fig. 3c). Tissue inhibitors of metalloproteinase, TIMP3 and TIMP4, were also downregulated in TGF-β2-treated BM-MSCs, although expression of TIMP1 and TIMP2 was unaffected by TGF-β2 treatment. LOX and LOXL2, which encode lysyl oxidase family members and are involved in cross-linking of collagen and elastin, had high levels of transcript detected in BM-MSCs and were significantly upregulated in TGF-β2-treated BM-MSCs. The timing of expression and coordination between secreted ECM subunits and enzymes that contribute to tendon ECM remodeling, maintenance, and healing are not well understood, so it is still unclear how significantly the changes in these secreted factors might affect the healing tendon.

### Cytokines and growth factors are differentially expressed in TGF-β2-treated BM-MSCs

**Fig. 4:**
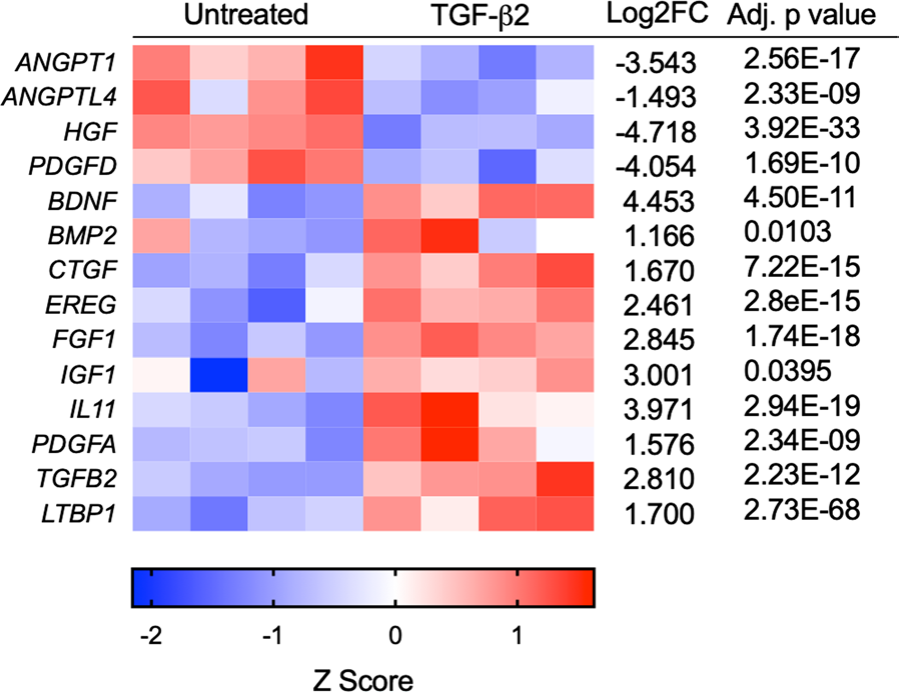
Cytokines and growth factor genes expressed in equine MSCs. Heatmap representation of cytokine and growth factor genes from RNA-sequencing for untreated and TGF-β2-treated equine MSCs. Data shown are Z scores, log_2_ fold change, and the adjusted *p* value for each gene for n = 4

Cytokines and growth factors regulate tendon healing by stimulating angiogenesis, extracellular matrix production, tenocyte proliferation, and modulating immune responses. Gene expression of several important cytokines and growth factors relevant to tendon healing was upregulated in TGF-β2-treated BM-MSCs compared to untreated controls (Fig. 4). Cytokines with the greatest upregulation in TGF-β2-treated BM-MSCs included bone-derived neurotropic factor (BDNF), interleukin-11 (IL-11), insulin-like growth factor-1 (IGF-1), and acidic fibroblast growth factor (FGF1). We attempted to measure IGF-1 protein expression from BM-MSCs using a commercial anti-human IGF-1 ELISA kit and an anti-human IGF-1 antibody in a western blot, but no protein could be detected (data not shown). Human IGF-1 and equine IGF-1 have 98% homology, but high levels of transcripts of five IGF binding proteins, IGFBP2, IGFBP4, IGFBP5, IGFBP6, and IGFBP7, were also detected in untreated and TGF-β2-treated BM-MSCs. IGFBPs can block antibody binding to IGF-1, which may have affected our ability to measure protein expression in cell culture supernatant and cell lysate [31, 32]. ANGPT1 and ANGPTL4, which may promote angiogenesis and tenocyte migration and cell cycle progression in injured tendons [33, 34], were downregulated in TGF-β2-treated BM-MSCs, but VEGFA, VEGFB, and VEGFC isoforms were not differentially expressed. PGE2 and TGF-β1 have been identified in the horse as the primary cytokines responsible for immunomodulation [35], and these were not differentially expressed in TGF-β2-treated BM-MSCs. This is supported by our previous work that did not find any significant difference in the concentrations of PGE2 and TGF-β1 in modified one-way mixed leukocyte reactions with untreated and TGF-β2-treated BM-MSCs [14].

### BM-MSCs increase tenocyte migration

**Fig. 5:**
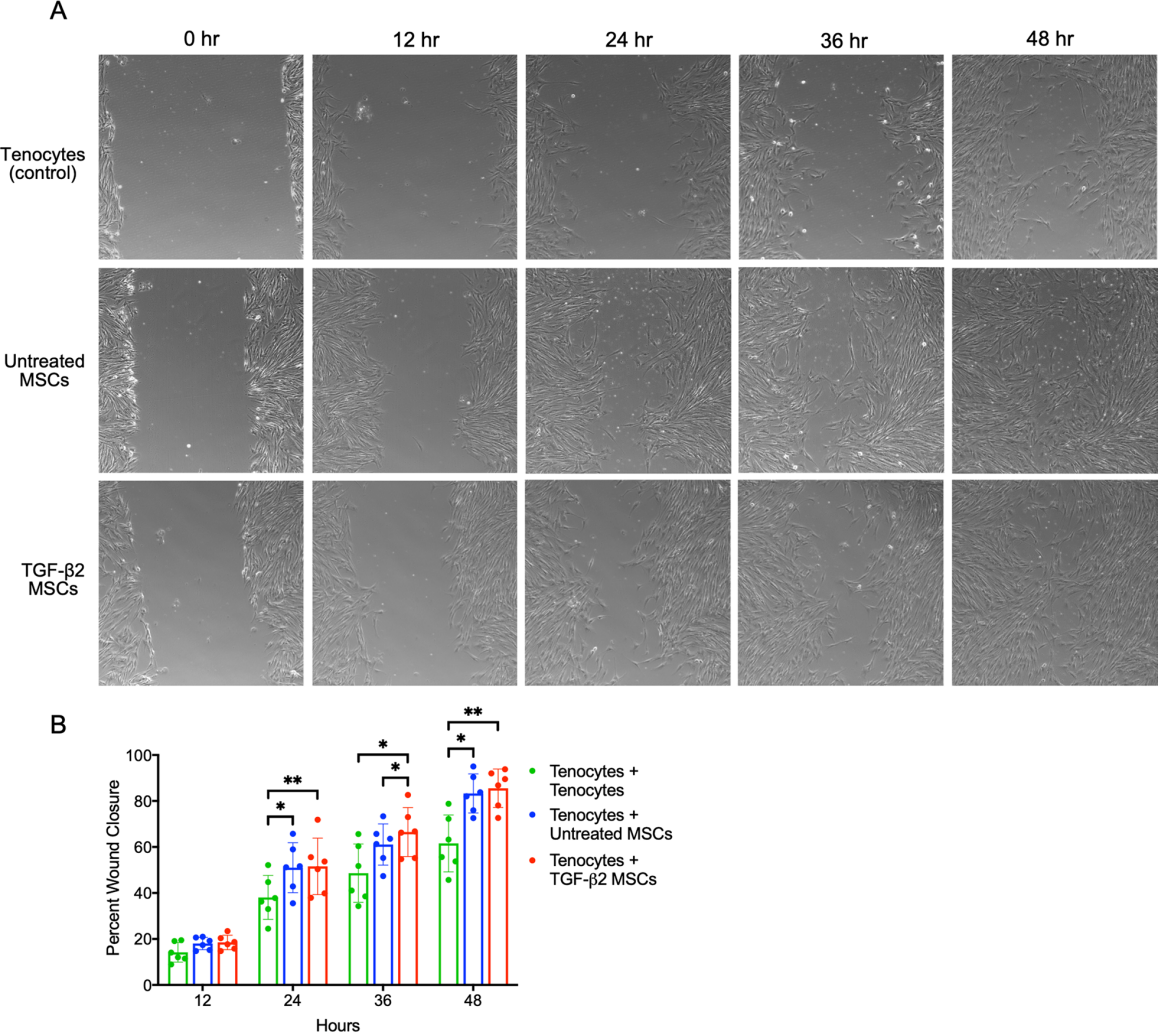
Tenocyte migration in MSC co-culture. **a** Representative images of the tenocyte scratch wound at 0, 24, 36, and 48 h post-scratch for tenocytes + tenocytes (control), tenocytes + untreated MSCs, and tenocytes + TGF-β2-treated MSCs. **b** The percent tenocyte wound closure at each time point for each treatment group. Data shown are mean ± SD for n = 6, *p < 0.05 and **p < 0.01 by a one-way repeated measures ANOVA with a Tukey’s post hoc test

Migration of tenocytes to the site of tendon damage following injury is an important initial step in the repair process [36]. To determine if the changes in the secretome of TGF-β2-treated BM-MSCs affect tenocyte migration, untreated and TGF-β2-treated BM-MSCs were indirectly co-cultured with tenocytes in a standard scratch assay. Tenocyte scratch wound closure was significantly improved with untreated BM-MSCs at 24 and 48 h and with TGF-β2-treated BM-MSCs at 24, 36, and 48 h (Fig. 5a). A significant difference between untreated and TGF-β2-treated BM-MSC groups was measured only at 36 h (Fig. 5b). An effect on tenocyte migration was noted between BM-MSCs from different horses (*p* < 0.0001), which is consistent with the known variation in the quantity of paracrine factors secreted from outbred species like humans and horses [37, 38]. Based on these results, the secretome of TGF-β2-treated BM-MSCs does not appear to be detrimental to tendon migration following wounding in an in vitro scratch wound assay.

## Discussion

Treatment of tendon injuries is a major challenge in human and veterinary orthopedic medicine, and the goals of regenerative therapies are to improve the tendon biochemical composition and biomechanical strength while reducing clinical reinjury rates. Advances in the understanding of MSC biology have led us to explore modulating cell function in an effort to improve MSC therapy for tendon injuries. In this study, we investigated changes in gene and protein expression of paracrine factors and ECM components related to tendon healing in equine BM-MSCs treated with TGF-β2. Using RNA-sequencing, we identified gene expression of numerous secreted factors important for tendon healing including COL1A1 and COL1A2, TNC, LOX, IGF-1, and FGF1 which were upregulated in TGF-β2-treated BM-MSCs compared to untreated MSCs. Further, when grown together in co-culture, both untreated and TGF-β2-treated BM-MSCs enhanced migration of equine tenocytes.

The clinical outcome of MSC therapy is dependent on both the quality and viability of the cells [39–42]. As seen in both the RNA-seq results and western blots, there were significant differences in the production of paracrine factors and ECM components between individual horses. This is consistent with reports that the quality of autologous MSCs can be affected by individual genetic backgrounds, health status, and age [43–45]. As shown in this study, the quantity of paracrine factors important for tendon healing can be increased by treating BM-MSCs with TGF-β2. Additionally, BM-MSCs from older horses may grow more slowly, but treating BM-MSCs with TGF-β2, provided bFGF is also included in the culture media, increases the yield of cells even from older horses [17]. Treating BM-MSCs with TGF-β2 could therefore potentially improve the efficacy of autologous therapy from patients who might otherwise be less than ideal candidates for MSC therapy including older patients. TGF-β2 also reduces MHC class I expression on MSCs, which reduces the cell-mediated immunogenicity of allogeneic BM-MSCs [14, 17]. In this study, we used different horses across experiments and still saw similar increases in both gene and protein expression of type I collagen and tenascin-c in the TGF-β2-treated MSCs. Even though BM-MSCs were treated with TGF-β2 for a much shorter period of time than the BM-MSCs used for RNA-seq, both type I collagen and tenascin-c protein expression was significantly upregulated. This is consistent with previously published in vitro and in vivo studies that have shown significant improvements in wound healing or clinical signs following treatment with MSCs licensed with cytokines for 24 or 48 h [40, 46–48]. Therefore, a short duration treatment of TGF-β2 is likely sufficient to significantly enhance the secretome of BM-MSCs and improve the efficacy of both autologous and allogeneic BM-MSC therapy, although more studies are needed to confirm this.

Type I collagen is the primary collagen of healthy tendon ECM. With aging and injury, the ECM contains higher levels of type III collagen along with more disorganized fibers [49]. Therefore, a strategy to improve tendon healing would include a shift to decrease deposition of type III collagen and concurrent remodeling into type I collagen. In western blots, TGF-β2-treated BM-MSCs had increased expression of the pro-alpha-1 chain and procollagen type I carboxy-terminal propeptide (PICP), which can only originate from synthesized type I collagen and is used as a marker of type I collagen production [50]. Increased PICP in the BM-MSC cultures may be due to both upregulation of type I procollagen and BMP1, which cleaves the carboxy terminus of type I procollagen [51]. BMP1 was upregulated in TGF-β2-treated BM-MSCs, although the log2 fold change was slightly less than 1. TGF-β2 treatment also enhanced BM-MSC gene expression of two of the three type V collagen alpha chains, which are incorporated into the type I collagen triple helix during collagen maturation in tendon tissue [22]. Tendon ECM development may be further supported through BM-MSC secretion of tenascin-C, which has been shown to enhance tendon stem cell migration and remodeling and accelerate restoration of tissue matrix after exogenous administration [52]. Therefore, TGF-β2-treated BM-MSCs may improve the composition of the tendon ECM during healing through direct secretion of both the collagenous and non-collagenous tendon ECM.

While many of the secreted factors upregulated following treatment could impart benefit to a healing tendon, others may be associated with fibrosis under certain conditions. IL-11 is a poorly understood pleiotropic cytokine belonging to the IL-6 family which plays a role in stromal cell proliferation, tissue healing and homeostasis, as well as fibrosis, but its role in tendon injury and repair is not well characterized [53–55]. Like TGF-β, BMP-2 drives extracellular matrix expression and can promote fibrosis in chronic tendon inflammation, although it also promotes tenocyte migration [56, 57]. HGF, an inhibitor of myofibroblast differentiation and TGF-β-induced fibrosis [58], is downregulated in TGF-β2-treated BM-MSCs. Due to the inherent complexity of biologic systems, the timing and cell specificity of cytokine and cytokine receptors involved in fibrosis, and our minimal understanding of what is required for a tendon to remodel to its native composition and strength [59], there are still many unknowns about the full effects of TGF-β2-treated BM-MSCs on tendon healing. Further research into how TGF-β2-treated BM-MSCs affect tenocyte gene expression and function is necessary to better predict any potential adverse effects. Additionally, while we do not see any overt morphologic changes in the BM-MSCs after adding TGF-β2 [17], in vivo testing in preclinical animal models is needed to confirm that TGF-β2-treated BM-MSCs do not promote ectopic bone or cartilage formation as some of the upregulated secreted factors are also involved in osteo-chondrogenic development.

Co-culture assays with BM-MSCs and tenocytes demonstrated that BM-MSC paracrine factors enhanced tenocyte migration. Other studies have established the role of migration in improved tendon healing [36, 57] and that MSC paracrine factors enhance cellular migration and proliferation of tenocytes as well as corneal epithelial cells and dermal fibroblasts [60–62]. Chen et al. determined that this tenocyte-specific response in improved cell migration was due to activation of the ERK1/2 pathway by MSC paracrine factors, although the exact factors responsible were not elucidated [60]. FGF1, TGF-β2, and IL-11, all which were upregulated in TGF-β2-treated BM-MSCs, can activate MAP kinase signaling as can angiotensin, prostaglandins, and platelet derived growth factors [63–66]. In this study, both untreated and TGF-β2-treated BM-MSCs significantly improved migration of tenocytes. While TGF-β2-treated BM-MSCs only showed an enhanced effect on migration over untreated BM-MSCs at a single time point, 36 h, it is not clear from this in vitro study if this difference would significantly enhance healing in vivo. However, migration is a single outcome measure and future studies investigating if TGF-β2-treated BM-MSCs improve tendon healing will focus more in depth on modulation of tenocyte function including ECM production and secretion of paracrine factors.

Genome-wide transcriptional analysis of equine BM-MSCs has not been extensively investigated despite the horse being an important large animal translational model for testing MSC regenerative therapies in musculoskeletal injuries [67, 68]. Harman et al. used single-cell RNA-seq to compare equine MSCs between tissue sources, but the focus of the reported data was on differences in phenotypic markers and expression of C-X-C motif chemokine ligand 6 (CXCL6) [69]. Although some cell functions can be inferred across species, differences in MSC phenotype and function have been previously identified in the horse including differences in important immunomodulatory factors [38, 70, 71]. Through RNA-sequencing, we revealed which secreted factor genes relevant to tendon healing are and are not expressed in equine BM-MSCs allowing for future comparisons between other species including humans and mice. We have also shown that TGF-β2 enhances gene expression of collagens, ECM matrix proteins, matrix remodeling enzymes, and growth factors in BM-MSCs. MSCs licensed with inflammatory cytokines typically have significant increases in immunomodulatory factors rather than collagen and other ECM-related proteins. As immunomodulatory factors play a critical role in tissue healing, treating BM-MSCs with both TGF-β2 and an inflammatory cytokine prior to treatment may enhance the paracrine function of MSCs in additional ways that are relevant to treatment of tendon injuries and should be further investigated. One limitation of this study is that it focuses primarily on the changes in gene expression in BM-MSCs following TGF-β2 treatment. However, the results from the RNA-seq do give us a better understanding of what proteins equine BM-MSCs potentially secrete, which allows us to better plan future experiments to characterize the entire secretome. Additional research into the changes in protein expression of BM-MSC secreted factors is also necessary to understand mechanisms of BM-MSC-mediated tissue repair and the differences in the BM-MSC secretome between the horse and the human for translational relevance.

## Conclusion

In this study, we demonstrated that treating equine BM-MSCs with TGF-β2 significantly increased production of secreted factors associated with tendon healing and maintained the ability of BM-MSCs to promote tenocyte migration. Increasing the expression of growth factors, collagens, and other extracellular matrix molecules from BM-MSCs by treating the cells with TGF-β2 prior to in vivo use may improve the consistency of secreted factor production and cell quality. More in vitro research into which BM-MSC secreted factors are most important for improving tendon healing is still needed as are preclinical animal models to investigate if manipulation of the secretome of BM-MSCs using TGF-β2 results in enhanced therapeutic efficacy for tendon injury.

## Supplementary Information

**Additional file 1**. Figure S1. GO terms, adjusted p value, and -log10(adjusted p value) for the top 200 upregulated genes in TGF-β2-treated BM-MSCs.

## Data Availability

The raw sequence data and normalized cells counts reported in this paper have been deposited in the Gene Expression Omnibus (GSE207394). All other data are available upon reasonable request.

## Abbreviations

BP: Biological processes
CC: Cellular components
ECM: Extracellular matrix
GO: Gene ontology
KEGG: Kyoto encyclopedia of genes and genomes
MF: Molecular function
MHC: Major histocompatibility complex
MSC: Mesenchymal stem cell
PICP: Procollagen type I carboxy-terminal propeptide
SDFT: Superficial digital flexor tendon
TGF-β: Transforming growth factor-β
