# Supplementary figures and images for: TGF-β2 enhances expression of equine bone marrow-derived mesenchymal stem cell paracrine factors with known associations to tendon healing
Source: Stem Cell Res Ther. 2022 Sep 16;13:477. doi: 10.1186/s13287-022-03172-9 (PMC9482193; doi:10.1186/s13287-022-03172-9)

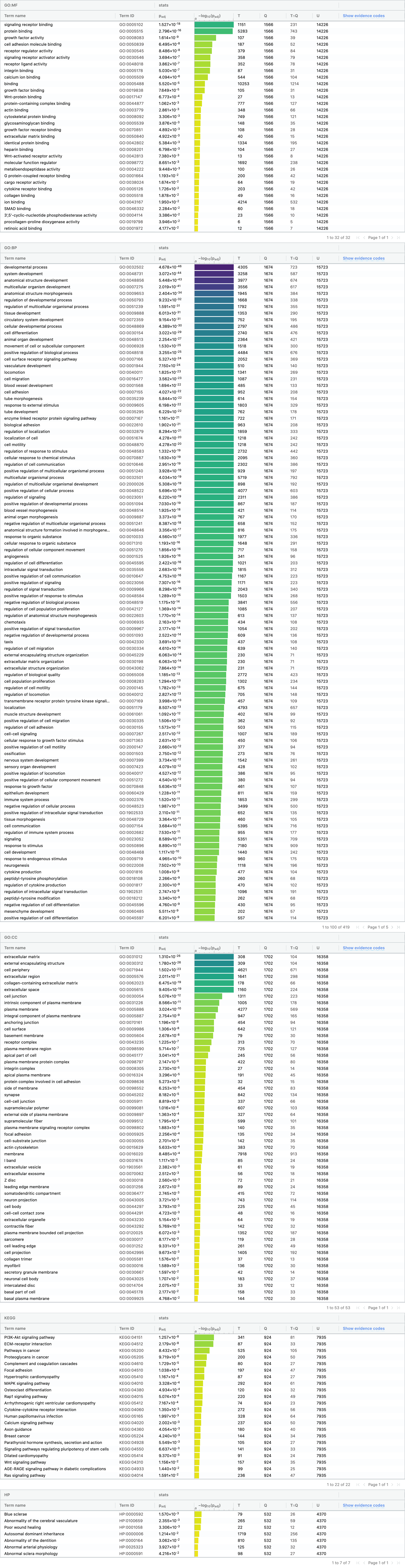

Supplement: Supplementary file 1 — Additional file 1. Figure S1. GO terms, adjusted p value, and -log10(adjusted p value) for the top 200 upregulated genes in TGF-β2-treated BM-MSCs. [file 13287_2022_3172_MOESM1_ESM.png]
